# Cardiorespiratory Dynamics as a Non-Autonomous System of Coupled Oscillators with Time-Varying Frequency Modulation

**DOI:** 10.3390/e28060685

**Published:** 2026-06-13

**Authors:** Hannah Brimble, Philip T. Clemson, Aneta Stefanovska

**Affiliations:** School of Physics and Astronomy, Lancaster University, Lancaster LA1 4YB, UK; hannah.brimble@btinternet.com (H.B.); p.t.clemson1@lancaster.ac.uk (P.T.C.)

**Keywords:** cardiorespiratory coupling, non-autonomous oscillators, phase synchronisation, time-varying dynamics, finite-time Lyapunov exponents, instantaneous attractors, respiratory modulation

## Abstract

We model the cardiorespiratory interaction as arising within a collection of coupled, non-autonomous, nonlinear oscillators with explicitly time-dependent frequency modulation. The resulting system is analysed in terms of phase tracking and stability using finite-time Lyapunov exponents. We show that synchronisation emerges from the interplay between coupling strength, intrinsic frequency mismatch, and modulation amplitude, giving rise to regimes of stable entrainment, intermittent synchronisation, and desynchronised dynamics. The transitions between these regimes are governed by the system’s ability to track time-dependent attractors rather than by fixed phase-locking conditions. Numerical simulations, together with physiological recordings, demonstrate that time-varying modulation and interaction structure are both essential to reproduce observed cardiorespiratory behaviour. In particular, the data indicate that coupling is not stationary but evolves over time, contributing significantly to the observed variability in synchronisation patterns. These results suggest that the cardiorespiratory interaction is more naturally interpreted as an emergent property of a non-autonomous dynamical system with evolving interaction geometry and moving attractors, rather than as a stationary coupling process between autonomous oscillators.

## 1. Introduction

The cardiovascular and respiratory systems form a coupled system of nonlinear oscillators whose interaction is inherently time-dependent and mediated through multiple physiological pathways. For many decades, the cardiovascular system has served as an important platform for the development of physical descriptions of living systems [1,2]. Early physiological control models focused primarily on haemodynamic regulation and cardiovascular control mechanisms [1,2,3,4], while later nonlinear and oscillator-based approaches introduced dynamical descriptions of cardiovascular variability and rhythmic interaction [5,6,7]. These modelling approaches successfully reproduced important static or time-averaged properties such as blood pressure, heart rate, and respiratory rate. However, such descriptions do not fully capture the dynamical structure of the cardiorespiratory interaction, which is characterised by continuous modulation, coupling, and variability across multiple temporal scales.

A prominent manifestation of the cardiorespiratory interaction is respiratory sinus arrhythmia (RSA), in which respiration modulates cardiac rhythm. RSA has been studied for centuries [8,9], yet its physiological origin and underlying mechanisms remain incompletely understood and continue to be debated [10]. Experimental evidence indicates that RSA emerges from the interaction of multiple physiological processes [11,12,13] operating across coupled mechanical, neural, and vascular scales, including respiratory gating of cardiac vagal outflow [14], and central autonomic coupling mechanisms [10]. Rather than acting as independent pathways, these processes form a tightly coupled feedback architecture in which respiratory, cardiovascular, and autonomic dynamics mutually constrain one another. From this perspective, RSA reflects an emergent property of distributed physiological coupling rather than a single dominant mechanistic pathway. Beyond its physiological significance, RSA is widely used as a non-invasive marker of autonomic regulation, cardiovascular ageing, and physiological adaptability [15,16].

From the perspective of nonlinear dynamics, the cardiorespiratory interaction can be interpreted as a system of coupled self-sustained oscillators [17]. Early large-scale modelling efforts, particularly those of Stefanovska and collaborators [18,19,20], described physiological rhythms in terms of interacting nonlinear oscillators. Accordingly, many observed features of cardiorespiratory variability can be understood in terms of coupling, phase interaction, and synchronisation phenomena. Related developments in physiological synchronisation theory further strengthened this interpretation [21,22,23,24,25].

These formulations were developed primarily within autonomous or weakly perturbed settings. In an autonomous dynamical system, the governing equations do not depend explicitly on time, and all variability must arise either from the internal state of the system or from externally imposed stochastic perturbations. Such formulations are widely used in nonlinear physiology and have been applied to a broad range of physiological signals. However, they do not provide an adequate description of systems operating under continuously evolving regulatory conditions.

Physiological systems are influenced by autonomic activity, metabolic demand, posture, and their environment [26]. Consequently, cardiorespiratory rhythms evolve around operating states that themselves vary in time. From this perspective, physiological variability need not be interpreted solely as stochastic forcing [20] or deterministic chaos [21,22], but may instead reflect structured temporal modulation of the governing dynamics [27].

These considerations naturally motivate modelling in terms of the use of non-autonomous dynamical systems, in which the governing equations depend explicitly on time [28,29]. Accordingly, temporal modulation is treated as an inherent component of the dynamics rather than as an external perturbation. Recent developments in nonlinear dynamics have extended oscillator theory into this regime, particularly through the theory of chronotaxic systems and related formulations of time-varying frequency oscillators [30,31,32,33,34,35,36,37,38]. Such systems possess time-dependent attractors and may nevertheless exhibit stable behaviour under continuous temporal modulation. This naturally captures physiological oscillators whose operating regimes evolve in time.

A synchronisation-based theoretical description of the cardiorespiratory interaction has also been proposed, in which RSA is formulated in terms of nonlinear entrainment and phase-locking between respiratory and cardiac oscillations [39]. This approach provides insight into coordinated rhythmic behaviour, particularly in terms of phase relationships and synchronisation stability. However, it is developed within a stationary or weakly time-dependent setting and does not explicitly address synchronisation in the presence of strongly non-autonomous dynamics.

An important consequence of introducing explicit time dependence is that stability can no longer be understood solely in terms of convergence to fixed states. Instead, stable behaviour must be defined relative to evolving dynamical structures. In this setting, synchronisation is not determined only by frequency matching or static phase locking, but also by the ability of the system to continuously track a time-dependent attractor. This distinction is essential in physiological systems, where regulatory processes operate continuously across multiple temporal scales and under non-stationary conditions.

Mathematical models of the cardiovascular and cardiorespiratory systems have therefore played an important role in bridging nonlinear dynamics and physiology, contributing substantially to the understanding of RSA and its implications for health [16,40,41,42]. In the present work, we reformulate RSA within a non-autonomous dynamical systems approach. Building on previous oscillator-based descriptions of cardiorespiratory coupling, we develop a model in which the cardiac oscillator evolves under explicitly time-dependent respiratory modulation. We then analyse the resulting dynamics in terms of synchronisation, moving attractors, and stability, and investigate how stable phase relationships emerge in the presence of continuous temporal modulation.

## 2. Autonomous and Non-Autonomous Models of Cardiorespiratory Dynamics

Cardiorespiratory dynamics is commonly described using coupled oscillator models, in which the heart and respiratory systems are represented as interacting self-sustained nonlinear oscillators [17,19]. The central idea of this class of models is that the observed macroscopic dynamics can be captured in terms of internal oscillator states and their mutual interactions, without explicit dependence on external time.

In this formulation, and using a phase–amplitude decomposition, a general autonomous representation of the cardiorespiratory interaction can be written as

(1) $$\begin{array}{cc} \frac{d\phi_{h}}{dt} & =\omega_{h}+f_{s}\left(\phi_{h}\right)+f_{c}\left(\phi_{h},R,\phi_{r}\right),\\ \frac{dR}{dt} & =A_{r}+G_{s}\left(R\right)+G_{c}\left(\phi_{h},R,\phi_{r}\right),\\ \frac{d\phi_{r}}{dt} & =\omega_{r}+g_{s}\left(\phi_{r}\right)+g_{c}\left(\phi_{h},R,\phi_{r}\right), \end{array}$$

where $\phi_{h}$ and $\phi_{r}$ denote the cardiac and respiratory phases, respectively, *R* represents the respiratory amplitude, and $\omega_{h}$ and $\omega_{r}$ are the intrinsic cardiac and respiratory frequencies. The functions $f_{s}$, $G_{s}$, and $g_{s}$ describe intrinsic oscillator dynamics, while $f_{c}$, $G_{c}$, and $g_{c}$ encode coupling between the components.

Within this formulation, RSA arises primarily through the coupling term $f_{c}$, which modulates cardiac phase evolution in response to respiratory dynamics. This class of models has been widely used for both phenomenological descriptions and data-driven reconstruction of physiological coupling structure [23,25].

Related theoretical studies have further examined how respiratory modulation can be disentangled from intrinsic cardiac variability within coupled oscillatory descriptions of RSA and heart-rate variability [43,44]. These works highlight the nontrivial dynamical structure underlying respiratory modulation of cardiac rhythms and provide additional support for oscillator-based interpretations of the cardiorespiratory interaction.

Physiological regulation is continuously influenced by slowly varying factors such as autonomic state, metabolic demand, posture, and environmental conditions, which act as structured modulations of intrinsic frequencies, amplitudes, and coupling strength.

This motivates a more general class of models in which explicit time dependence is introduced, leading to non-autonomous dynamical systems [28]. The autonomous formulation is extended to

(2) $$\begin{array}{cc} \frac{d\phi_{h}}{dt} & =\omega_{h}\left(t\right)+f_{s}\left(\phi_{h},t\right)+f_{c}\left(\phi_{h},R,\phi_{r},t\right),\\ \frac{dR}{dt} & =A_{r}\left(t\right)+G_{s}\left(R,t\right)+G_{c}\left(\phi_{h},R,\phi_{r},t\right),\\ \frac{d\phi_{r}}{dt} & =\omega_{r}\left(t\right)+g_{s}\left(\phi_{r},t\right)+g_{c}\left(\phi_{h},R,\phi_{r},t\right), \end{array}$$

where intrinsic frequencies, amplitudes, and coupling functions may vary explicitly in time. The system evolves on a time-dependent dynamical landscape, so that stability and synchronisation must be interpreted relative to evolving phase-space structures rather than to fixed attractors.

Synchronisation corresponds to the ability of trajectories to remain bounded relative to time-dependent dynamical constraints. This relates naturally to non-autonomous and chronotaxic oscillator theory, where stable behaviour may persist under explicit temporal modulation [31,32,33,35,36,38].

The following section introduces a minimal oscillator model that captures these effects, focusing on the role of time-dependent modulation in shaping synchronisation and stability in the cardiorespiratory interaction.

## 3. The Non-Autonomous Model

This section introduces the non-autonomous oscillator model and investigates its dynamical behaviour across relevant parameter regimes. Stability is characterised using Lyapunov exponents, and the resulting dynamics is analysed in both the time and time–frequency domains.

### 3.1. Model Formulation

We describe the cardiorespiratory interaction using a phase oscillator model incorporating phase modulation, motivated by [7,35]. The cardiac dynamics is given by

(3) $$\frac{d\phi_{h}}{dt}=\omega_{h}+\gamma \sin\left(\phi_{h}-\phi_{1}\right),$$

where $\phi_{h}$ denotes the phase of the cardiac cycle, and $\omega_{h}$ is the intrinsic cardiac frequency in the absence of external modulation. The second term represents coupling to a driving phase $\phi_{1}\left(t\right)$ with strength γ.

This formulation treats the heart as a self-sustained oscillator whose phase is modulated by an external rhythmic input. In RSA, $\phi_{1}\left(t\right)$ represents the effective respiratory drive acting on cardiac timing.

The dynamics of the driving phase is defined as

(4) $$\frac{d\phi_{1}}{dt}=\omega_{1}(1+kf(\omega_{m},t)),$$

where $\omega_{1}$ is the baseline frequency of the driving signal. The function $f(\omega_{m},t)$ is a bounded periodic modulation with values in [−1, 1] and characteristic frequency $\omega_{m}$, while *k* determines the modulation amplitude.

Physiologically, $f(\omega_{m},t)$ represents the respiratory cycle. In the present simplified formulation, we take

$$f\left(\omega_{m},t\right)=\sin\left(\omega_{m}t\right),$$

so that respiration is represented as a single periodic modulation of the driving frequency. With this choice, Equation (4) integrates to

(5) $$\phi_{1}\left(t\right)=\omega_{1}\left[t-\frac{k}{\omega_{m}}(\cos(\omega_{m}t)-1)\right],$$

showing that the respiratory drive corresponds to a time-modulated phase evolution. Identifying $\omega_{m}\equiv \frac{d\phi_{r}}{dt}$, the modulation can alternatively be expressed in terms of respiratory phase as

(6) $$\phi_{1}=\omega_{1}\left[t-\frac{k}{\omega_{m}}(\cos(\phi_{r})-1)\right],$$

linking the formulation to the general non-autonomous description introduced earlier.

A key feature of Equation (3) is that the cardiac oscillator retains a non-zero intrinsic frequency $\omega_{h}$, reflecting its self-sustained nature. At the same time, the system is non-autonomous due to the explicit time dependence of $\phi_{1}\left(t\right)$.

### 3.2. Stability and Synchronisation

To interpret the dynamics of the system, we must clarify how stability and synchronisation are defined in a non-autonomous setting. The central requirement is that the cardiac oscillator maintains bounded behaviour relative to a time-dependent driving signal.

#### 3.2.1. Phase Dynamics and Limit Cycle Representation

The cardiac oscillator can be represented as motion along a limit cycle in phase space. The phase $\phi_{h}$ corresponds to the angular coordinate of this motion, defining the instantaneous state of the oscillator.

#### 3.2.2. Instantaneous Attractor

Coupling to respiration introduces a phase-dependent deformation of the limit cycle dynamics. At each instant, there exists a preferred phase toward which trajectories are attracted. This defines an *instantaneous attractor* in phase space.

Because the system is non-autonomous, this attractor evolves in time. The observed dynamics arises from the competition between attraction toward this instantaneous structure and its temporal motion.

#### 3.2.3. Synchronisation Regimes

The qualitative behaviour of the system depends on whether the evolving attractor can be tracked:

- If a stable attractor exists at all times and is dynamically tracked, the system exhibits stable synchronisation.
- If the attractor exists intermittently, the system shows intermittent synchronisation.
- If no attractor exists, the system cannot maintain phase locking.

Thus, synchronisation is defined not only by frequency alignment but by bounded phase evolution relative to a time-dependent attractor.

### 3.3. Quantifying Stability: Lyapunov Exponents

To quantify stability, we use Lyapunov exponents, which measure the growth or decay of infinitesimal perturbations [45,46]. For two trajectories with initial separation $\delta q_{0}$, the evolution of the separation δq(t) defines the maximal Lyapunov exponent

(7) $$\lambda =\lim_{t\to \infty }\frac{1}{t}\ln\left(\frac{∥\delta q(t)∥}{∥\delta q_{0}∥}\right).$$

Finite-time Lyapunov exponents (FTLEs) are used in practice to characterise time-dependent stability [35]. The sign of λ determines stability:

- λ<0: stable dynamics with convergence to the evolving attractor,
- λ=0: neutral stability,
- λ>0: divergence and loss of synchronisation.

Negative Lyapunov exponents indicate successful tracking of the time-dependent attractor, while positive values correspond to breakdown of phase locking.

### 3.4. Dynamics in the Non-Autonomous Frame

**Figure 1:**
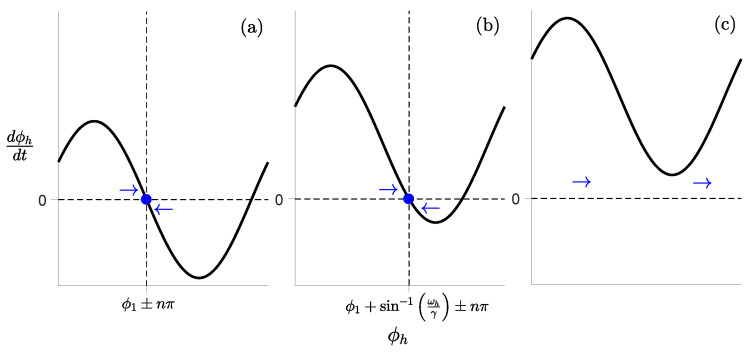
Phase dynamics $d\phi_{h}/dt$ as a function of $\phi_{h}$ at fixed $\phi_{1}$. Intersections with zero correspond to fixed points. Stable fixed points define instantaneous attractors. Panels show: (**a**) purely driven dynamics, (**b**) stable attractor regime, (**c**) loss of attractor. Arrows indicate direction of attraction.

To clarify the role of time dependence, it is useful to examine the system in an instantaneous (frozen) frame where $\phi_{1}$ is held fixed. In this representation, the system behaves as an autonomous oscillator with parameter-dependent dynamics. The phase evolution is governed by the balance between intrinsic frequency and coupling, as illustrated in Figure 1, which shows $d\phi_{h}/dt$ as a function of $\phi_{h}$.

When a stable fixed point exists, trajectories converge locally, corresponding to instantaneous phase locking. However, in the full system $\phi_{1}\left(t\right)$ evolves in time, so these attractors move through phase space. This leads to a crucial distinction: instantaneous stability does not imply global synchronisation. Instead, synchronisation requires that the system continuously tracks the moving attractor.

To quantify this effect, we define the instantaneous frequency mismatch

(8) $$\Delta \Omega \left(t\right)=\omega_{h}-\omega_{1}\left(1+kf(\omega_{m},t)\right)=\Delta \omega -k\omega_{1}f\left(\omega_{m},t\right),$$

with $\Delta \omega =\omega_{h}-\omega_{1}$. For k=0, the system reduces to a classical synchronisation problem with constant mismatch. For k>0, the mismatch becomes time-dependent, and the attractor undergoes periodic deformation. In this regime, synchronisation depends on whether the cardiac phase can continuously adapt to the evolving attractor.

**Figure 2:**
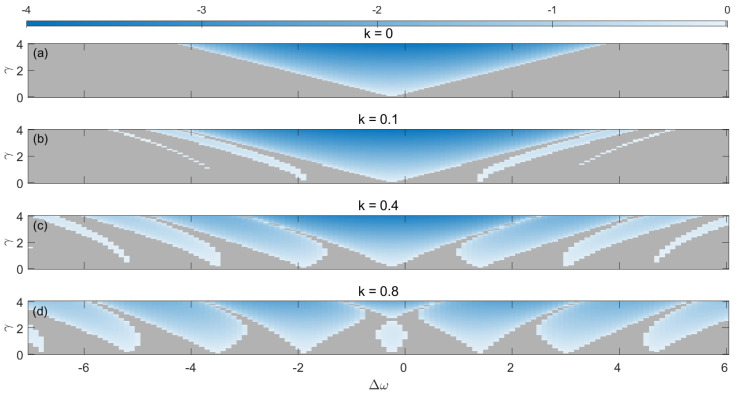
Maximum finite time Lyapunov exponents (FTLEs), λ, for different values of the mismatch in natural frequencies, Δω, for the system defined in Equation (3). The FTLEs were computed over 700 s. The parameter values were $\omega_{m}=0.5\pi$, $\omega_{1}=6$, and (**a**) k=0, (**b**) k=0.1, (**c**) k=0.4, (**d**) k=0.8. The blue regions correspond to the actual values of the Lypunov exponents and indicate areas of synchronisation.

Unlike previous studies assuming slow modulation [35], where the modulation frequency is small compared with the characteristic oscillator frequency ($\omega_{m}\ll \omega_{1}$), the present model allows modulation on timescales comparable to the cardiac rhythm. In the simulations presented here, $\omega_{m}=0.5\pi$ and $\omega_{1}=6$, giving $\omega_{m}/\omega_{1}\approx 0.26$, so the modulation cannot be regarded as asymptotically slow. The resulting dynamics therefore lies outside the regime where adiabatic approximations apply and must be studied numerically. Analytical treatment becomes difficult in this fully non-autonomous regime, and the system must be studied numerically. The representative dynamics is shown in Figure 2.

These results illustrate how synchronisation in cardiorespiratory dynamics can be a non-autonomous phenomenon governed by the ability to track time-dependent attractors rather than static phase relationships.

## 4. Numerical Analysis

In this section, we analyse the dynamics of the proposed non-autonomous oscillator model numerically. Since the system does not admit a general closed-form solution in the presence of time-dependent modulation, numerical integration is used to explore its behaviour across different parameter regimes. We focus in particular on how the interplay between intrinsic frequency mismatch, coupling strength, and respiratory modulation determines the emergence, loss, or intermittency of phase synchronisation between the cardiac and respiratory oscillators. A key point in the following analysis is that synchronisation is not treated as a purely static property, but as a dynamical regime characterised by stability of phase relations under continuous time-dependent modulation. This motivates the use of Lyapunov exponents, time-frequency analysis, and data-driven inference methods.

### 4.1. Lyapunov Exponents and Synchronisation

In order to determine the conditions under which synchronisation occurs for k>0, we analyse the stability of the coupled system using Lyapunov exponents. In the present context, synchronisation is understood as the emergence of frequency entrainment between the cardiac phase $\phi_{h}$ and the driving phase $\phi_{1}$, together with bounded phase differences over time. To make this notion precise, we consider the system in an instantaneous (autonomous) frame, where the slow time-dependence of the modulation is frozen at a given time $t_{0}$. This yields

(9) $$\begin{array}{cc} \frac{d\phi_{h}}{dt} & =\omega_{h}+\gamma \sin\left(\phi_{h}-\phi_{1}\right),\\ \frac{d\phi_{1}}{dt} & =\omega_{1}+\left(1+kf(\omega_{m},t_{0})\right), \end{array}$$

where $t_{0}$ is treated as a fixed parameter. In this frozen frame, synchronisation can be interpreted in the classical dynamical systems sense: if nearby trajectories converge, the system approaches a stable phase-locked state. This is quantified by the maximum FTLE. When the FTLE is negative, perturbations decay and trajectories converge towards a stable phase relation. In this regime, illustrated in Figure 1b, the phase difference converges to

$$\phi_{h}-\phi_{1}=\sin^{-1}\left(\frac{\omega_{h}}{\gamma }\right)\pm n\pi ,$$

and the two oscillators become frequency-entrained.

In the following analysis, we compute both long-time and time-localised FTLEs following the methodology in [35]. This allows us to distinguish persistent synchronisation from transient or intermittent stability. Throughout the simulations, we use $\omega_{m}=0.5\pi$ and $\omega_{1}=6$. This choice places the system outside the asymptotically slow-modulation regime ($\omega_{m}\ll \omega_{1}$) considered in earlier analytical studies [35], so the adiabatic approximation is no longer applicable and the dynamics must be analysed numerically.

Figure 2 shows the regions of synchronisation as identified by the maximum long-time FTLE as a function of frequency mismatch Δω and coupling strength γ. The classical Arnold tongue structure is recovered: increasing γ enlarges the region of synchronisation, allowing entrainment over progressively larger mismatches. For k=0, the results reproduce those of [35]. For k>0, however, the structure becomes richer: additional synchronisation regions appear at shifted values of Δω. These additional “tongues” are not present in the slowly modulated regime studied previously and arise from the fast modulation of the driving frequency.

The influence of oscillatory modulation on cardiorespiratory synchronisation has also been reported experimentally during both rest and exercise conditions [47], supporting the interpretation that synchronisation structure depends sensitively on time-varying physiological dynamics. Physically, rapid modulation can temporarily compensate for large frequency mismatches, allowing synchronisation even in parameter regimes where it would not be expected in a static system. Nevertheless, the overall trend remains consistent: increasing *k* enlarges the total region in which synchronisation is observed.

### 4.2. Time Series Analysis

#### 4.2.1. Time-Frequency Analysis and Ridge Extraction

In addition to stability analysis using FTLEs, we compute the continuous wavelet transform of the time series $x\left(t\right)=\sin\phi_{h}$ in order to characterise the time-dependent frequency content of the cardiac oscillator.

Time frequency analysis decomposes a signal into its constituent frequencies. At each moment in time, a wavelet, a function with finite energy and zero mean, is stretched or compressed. If the wavelet is a good match to the signal a large transform is assigned. The wavelet transform is defined by the convolution of the time series with the wavelet [48,49],

(10) $$W_{x}\left(t,f\right)=2\pi f\int_{-\infty }^{\infty }x\left(u\right)\;\psi \left(2\pi f(u-t)\right)\;du,$$

where x(u) is the signal, *f* is frequency, *t* is time, and ψ(·) is the wavelet.

The log-normal wavelet [49] is given by

(11) $$\psi \left(x\right)=\frac{1}{2\pi }\int_{-\infty }^{\infty }\hat{\psi }\left(y\right)e^{ixy}\;dy,$$

where $\hat{\psi }\left(y\right)$ is

(12) $$\hat{\psi }\left(y\right)=\left\{\begin{array}{cc} \exp\;\left(-\frac{{\left(2\pi f_{r}\log y\right)}^{2}}{2}\right), & y>0,\\ 0, & y\leq 0, \end{array}\right.$$

and where $f_{r}$ is the frequency resolution parameter [50]. An adaptable window size is used in order to optimise the time-frequency resolution at all frequencies.

From the time-frequency representation, the frequency at each time can be extracted using ridge extraction [50]. This selects the frequency at each instant in time with the greatest amplitude. This method is used to extract the instantaneous heart rate from both simulated and measured time-series data.

#### 4.2.2. Phase Coherence

Wavelet phase coherence characterises the consistency of the phase differences between two signals and provides an indicator of the existence of interactions. It is defined as [51,52,53,54]

(13) $$\rho \left(t,f\right)=\frac{1}{\delta }\left|\int_{t-\delta /2}^{t+\delta /2}e^{i\left(\phi_{a}\left(f,\tau \right)-\phi_{b}\left(f,\tau \right)\right)}\;d\tau \right|,$$

where $\phi_{a}\left(f,\tau \right)$ and $\phi_{b}\left(f,\tau \right)$ denote the instantaneous phases of the two signals obtained from the wavelet transform at frequency *f* and time τ, and δ is the width of the averaging window.

The quantity ρ(t,f) is bounded between 0 and 1. Values close to 1 indicate that the phase difference remains approximately constant over the averaging window, which is a necessary condition for phase synchronisation. Values close to 0 indicate rapidly varying phase differences and the absence of consistent phase relationships.

**Figure 3:**
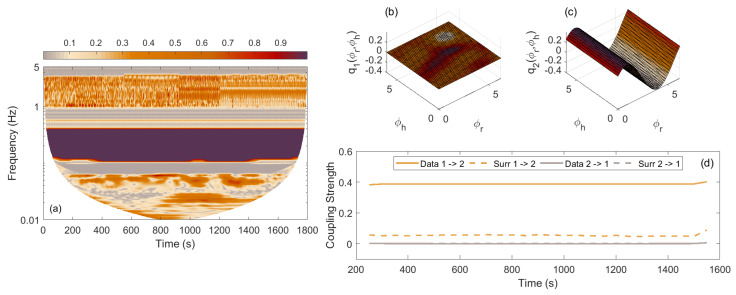
Coupled phase oscillator model with $\omega_{m}=0.5\pi$, $\omega_{1}=6$, k=0.1, γ=1.5. (**a**) Instantaneous phase coherence with frequency resolution 1. (**b**) Coupling function from HRV to respiration. (**c**) Coupling function from respiration to heart rate variability (HRV). Dark red indicates strong positive coupling amplitude, grey indicates strong negative coupling amplitude, and orange indicates weak or no coupling in either direction. (**d**) Coupling strength from respiration to HRV (orange line) and from HRV to respiration (grey line), with 95% surrogate significance thresholds indicated by dashed lines, confirming strong coupling from respiration to HRV.

Figure 3a shows phase coherence analysis for an example time series simulated using the cardiorespiratory model. The large purple band around the respiratory frequency interval corresponds to high phase coherence ρ≈1.

#### 4.2.3. Coupling Function Analysis

To compare the model with experimental data, we infer coupling functions using dynamical Bayesian inference [55]. We emphasise that the coupling function represents the full functional form of the phase-dependent interaction, whereas the coupling strength is a reduced summary statistic derived from it. In general, the coupling function determines the structure of interaction, while the coupling strength provides a scalar measure of its effective magnitude over a specified temporal interval [56,57].

### 4.3. Dynamical Bayesian Inference

Dynamical Bayesian inference is a method used to determine whether coupling exists between two time series and to estimate both the coupling strength and coupling functions in each direction [55,58,59,60]. It is applied here to infer the cardiorespiratory interaction from numerically generated and measured data.

The phase dynamics is written as

(14) $$\frac{d\phi_{i}}{dt}=\omega_{i}+f_{s,i}\left(\phi_{i}\right)+f_{c,i}\left(\phi_{i},\phi_{j}\right)+\xi \left(t\right),$$

where $\omega_{i}$, $f_{s,i}\left(\phi_{i}\right)$, and $f_{c,i}\left(\phi_{i},\phi_{j}\right)$ denote the intrinsic frequency, self-dynamics, and coupling terms, respectively, and ξ(t) is Gaussian white noise.

In dynamical Bayesian inference, the coupling functions are expanded on a truncated Fourier basis under the assumption of periodicity. The self-term is written as

(15) $$f_{s,i}\left(\phi_{i}\right)=\sum_{k=1}^{K}a_{i,2k}\sin\left(k\phi_{i}\right)+a_{i,2k+1}\cos\left(k\phi_{i}\right),$$

and the coupling term as

(16) $$f_{c,i}\left(\phi_{i},\phi_{j}\right)=\sum_{r=1}^{R}\sum_{s=1}^{S}b_{i,rs}\;e^{2\pi ir\phi_{i}}e^{2\pi is\phi_{j}}.$$

In practice, the sums are truncated to finite orders to avoid overfitting.

The Fourier coefficients are estimated using Bayes’ theorem in a recursive scheme, where data are analysed in time windows and the posterior from each window is used as the prior for the next. A coupling strength measure is obtained from the inferred phase dynamics, typically based on the variability of the phase derivative. Statistical significance is assessed using surrogate data as discussed in Section 4.5.

Figure 3b–d show the results of the dynamical Bayesian inference analysis of the simulated time series from the cardiorespiratory model. The amplitudes of the coupling functions in the two directions are shown in Figure 3b,c, with dark red indicating strong positive coupling amplitude, grey indicating strong negative coupling amplitude, and orange indicating weak or no coupling in either direction. Figure 3d shows the time-evolving coupling strength in both directions, with significant respiration → HRV coupling above the 95% surrogate level (orange line) and no significant HRV → respiration coupling (grey line).

### 4.4. Parameter Selection

#### 4.4.1. Selection of *k*

**Figure 4:**
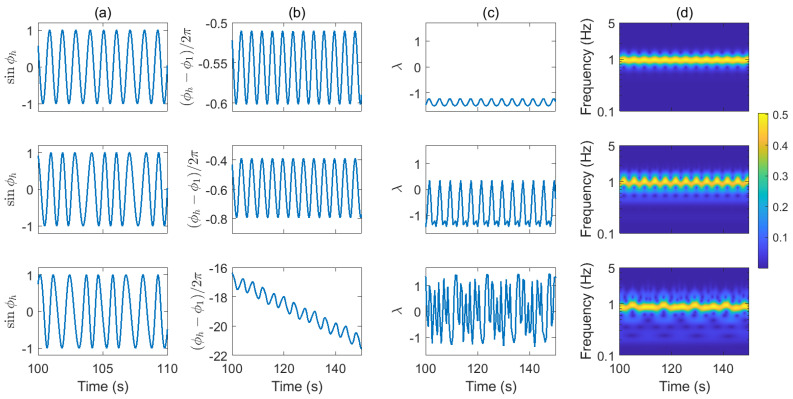
Results of numerical simulations of the system defined in (3) for different values of *k*. The top row shows the results for k=0.1, the middle row for k=0.4, and the bottom row for k=0.8. The plots in each column show: (**a**) a time series sample of $\sin(\phi_{h})$; (**b**) the phase difference between $\phi_{h}$ and $\phi_{1}$; (**c**) the maximum time-localised FTLE; (**d**) a time-frequency representation of $\sin(\phi_{h})$ via the wavelet transform amplitude |W| using the continuous wavelet transform with frequency resolution $f_{r}=1$.

Figure 4 shows results for different values of *k*. For k=0.1, the time series appears nearly stationary, although modulation is visible in phase differences, FTLE, and wavelet structure. For k=0.4, modulation is clearly visible directly in the time series, while FTLE becomes intermittently positive. For k=0.8, strong modulation leads to phase slips and loss of stable frequency structure. Thus, k=0.8 corresponds to an overly unstable regime, while k=0.1 under-represents non-autonomous effects. We therefore select k=0.4 as it provides a balance between strong modulation and dynamical stability.

#### 4.4.2. Selection of γ

**Figure 5:**
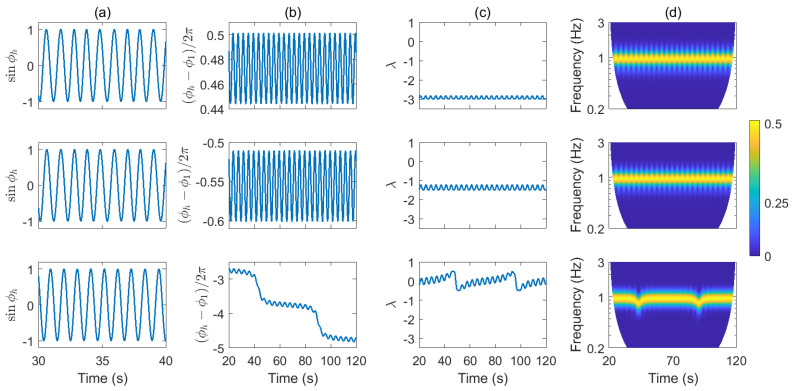
Results of numerical simulations of the system defined in (3) for different parameter values of properties of the coupled phase oscillator model with $\omega_{m}=0.5\pi ,\omega_{1}=6$, and k=0.1, over different γ. The top row shows the results for γ=3, the middle row for γ=1.5, and the bottom row for γ=0.5. The plots in each column show: (**a**) a time series sample of $\sin(\phi_{h})$; (**b**) the phase difference between $\phi_{h}$ and $\phi_{1}$; (**c**) the maximum time-localised FTLE; (**d**) the wavelet transform amplitude |W| of $\sin(\phi_{h})$ using the continuous wavelet transform with $f_{r}=1$. As γ decreases the system becomes less stable.

Figure 5 shows results for different coupling strengths. For γ=3, the system is strongly stable (λ≈−3). For γ=1.5, stability remains but sensitivity increases. For γ=0.5, intermittent phase slips occur and FTLE becomes positive at times, indicating loss of reliable entrainment. Thus, γ=3 and γ=1.5 represent physiologically meaningful regimes, while γ=0.5 leads to instability.

### 4.5. Statistical Significance Testing Using Surrogate Data

To assess whether observed coherence or coupling functions are statistically significant, surrogate data methods are commonly used [61,62]. This is necessary because even independent signals can exhibit non-zero coherence due to finite data length and coincidental alignment of fluctuations.

The null hypothesis of no coupling is tested by generating surrogate time series that preserve key statistical properties of the original data while removing any true interdependence. The same coherence, or coupling function analysis is then applied to both original and surrogate data, yielding a distribution of coherence values under the null hypothesis. Significance is determined by comparing the observed coherence to a chosen percentile of this distribution, typically the 95% threshold.

In this work, surrogate testing is used to distinguish genuine phase and coupling relationships from those arising by chance, thus ensuring that only statistically robust coherence features are interpreted as indicative of interaction.

## 5. Comparison with Measured Data

To assess whether the dynamical quantities introduced by the model can be identified in physiological recordings, we analyse cardiorespiratory signals obtained from two healthy male subjects aged 22 and 72 years during supine rest. An electrocardiogram (ECG) was recorded using a three-electrode configuration placed on the shoulders and lower left rib to optimize the detection of R-peak, and respiration was measured using a thoracic stretch-sensitive belt providing an estimate of tidal volume. The signals were sampled at 1200 Hz over 30 min and subsequently downsampled for analysis.

**Figure 6:**
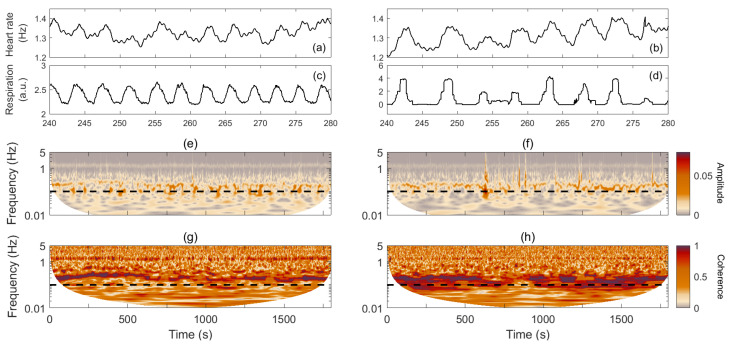
Dynamical properties of a young (**left**) and older (**right**) male subject. (**a**,**b**) Heart rate over time is obtained using ridge extraction. It is traditionally known as HRV, and is instantaneous frequency of the heart beat within the non-autonomous, time-localised dynamics. (**c**,**d**) Respiration signals. (**e**,**f**) Continuous wavelet transform of the HRV signals. (**g**,**h**) Phase coherence between the HRV and respiration signals. The black dashed lines in (**e**–**h**) indicate 0.1 Hz. All signals were obtained by downsampling from 1200 Hz to 48 Hz, with a frequency resolution of 1.

Cardiac and respiratory phases were extracted using ridge-based wavelet analysis [50]. The heart rate shown in Figure 6a,b is obtained using ridge extraction. It is known as HRV, or as the instantaneous frequency of the heart over time. This representation provides a time-resolved estimate of the instantaneous cardiac rhythm and enables direct comparison with the model variables $\phi_{h}$ and $\phi_{1}$.

Phase coherence, time–frequency representations, and coupling functions were then calculated in order to characterise the interaction between respiratory and cardiac dynamics. Dynamical Bayesian inference was additionally used to estimate coupling functions and coupling strengths in both directions.

Figure 6 shows representative cardiorespiratory recordings together with their time–frequency and phase-coherence characteristics. Several features observed in the model are also present in the measured data. Both subjects exhibit pronounced respiratory modulation of the cardiac rhythm, visible as time-dependent frequency variation in the HRV signal and as coherent structure in the wavelet representation. In addition, the coherence maps reveal that the interaction is not stationary but varies across time, indicating that the cardiorespiratory relationship evolves over the recording interval.

These observations are consistent with the central assumption of the model, namely, that cardiorespiratory dynamics is governed by time-dependent interactions rather than by fixed coupling parameters.

**Figure 7:**
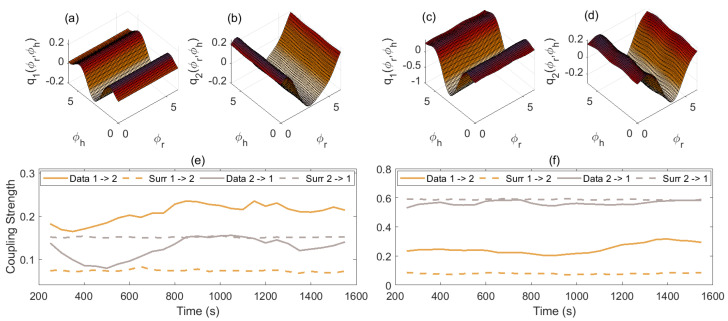
Dynamical Bayesian inference of measured data in two male subjects: a 22-year-old subject (**a**,**b**,**e**) and a 72-year-old subject (**c**,**d**,**f**). (**a**,**c**) Coupling functions from heart rate variability (HRV) to respiration. (**b**,**d**) Coupling functions from respiration to HRV. Dark red indicates strong positive coupling amplitude, grey indicates strong negative coupling amplitude, and orange indicates weak or no coupling in either direction. (**e**,**f**) Time-averaged coupling strengths from respiration to HRV (orange lime) and from HRV to respiration (grey line) with the 95% surrogate significance threshold indicated by dashed lines.

Figure 7 shows coupling functions inferred from the same recordings using dynamical Bayesian inference. In both subjects, a significant influence from respiration to HRV is observed, whereas the reverse direction remains weak and close to surrogate levels. This directional asymmetry is consistent with the respiratory modulation incorporated in the model.

The inferred coupling strengths are not constant in time but fluctuate throughout the recordings. This observation is important because the proposed model assumes that cardiorespiratory interaction evolves under explicitly time-dependent modulation. The measured data therefore support the view that cardiorespiratory dynamics cannot always be represented adequately by stationary coupling parameters.

The two subjects differ primarily in the temporal organisation of the interaction. The younger subject exhibits stronger temporal variability in the inferred coupling, whereas the older subject displays a comparatively more stationary interaction profile. The physiological significance of this observation is discussed in the following subsection in the context of previous population studies.

These observations correspond to different regimes of the effective coupling parameters. In the younger participant, the inferred coupling strength exhibits stronger temporal variation, resulting in continuous re-adjustment of phase relationships. In the older participant, the coupling varies weakly in time and remains approximately stationary over the recording interval. Importantly, both subjects remain within a regime of persistent respiration-to-heart entrainment, as indicated by bounded phase differences. The key difference is therefore not the existence of coupling, but its temporal variability. This supports the interpretation of synchronisation as a non-autonomous phenomenon in which physiological ageing is associated with reduced variability of coupling and decreased dynamical flexibility of cardiorespiratory coordination. This observation is consistent with the broader finding that physiological variability across cardiovascular processes tends to decrease with age [63].

The two subjects are selected from a previously studied cohort of healthy individuals used in a population analysis of cardiorespiratory interactions [16]. A detailed interpretation of these differences in the context of population-level ageing effects and previously established physiological findings is provided in Section 5.1.

### 5.1. Population Context and Relation to Previous Cohort Studies

The two subjects analysed here were selected from a larger cohort of healthy individuals previously studied in a systematic investigation of age-related changes in cardiorespiratory interactions [16]. That study examined participants spanning a broad age range using time–frequency analysis, phase coherence, and coupling inference methods, and established population-level trends in cardiorespiratory coordination across ageing.

The present work does not revisit those statistical analyses. Instead, the two recordings are used as representative examples drawn from the physiological regimes identified in the larger cohort. Both subjects were clinically healthy at the time of recording, so the observed differences are interpreted in the context of healthy ageing rather than pathology.

The younger subject is representative of the more dynamically variable interaction patterns observed in younger individuals, whereas the older subject is representative of the comparatively less variable interaction patterns observed at advanced age. The examples therefore provide a physiologically grounded reference against which the behaviour of the proposed non-autonomous model can be compared.

A detailed comparison between the model predictions and established physiological observations from the cardiorespiratory literature is provided in the following section.

### 5.2. Relation to Existing Physiological, Data-Driven, and Modelling Approaches

RSA and cardiorespiratory interactions have been investigated from physiological, signal-processing, and dynamical-systems perspectives for several decades. Early studies established the central role of respiration in shaping heart-rate variability and demonstrated that cardiorespiratory interactions cannot be understood solely in terms of average heart-rate modulation [64,65].

Transfer-function analyses further showed that the relation between respiration and heart-period variability depends on both frequency and phase, revealing a structured dynamical interaction between the two rhythms [66]. These descriptions are typically based on linear assumptions and local stationarity within analysis windows.

Subsequent investigations characterised RSA and cardioventilatory coupling using spectral, phase-based, and variability measures, highlighting both the usefulness and limitations of these standard analytical approaches for describing cardiorespiratory interactions [67,68,69,70,71].

RSA has been characterised using signal-processing and variability-based approaches. These studies quantified respiratory modulation with respect to respiratory phase, regularity, synchronisation, and temporal organisation [68,69,70,71]. More generally, they established that cardiorespiratory interactions vary across physiological conditions and are not captured by a single scalar measure. These approaches typically rely on time or cycle averaging and often assume linearity or local stationarity within analysis windows.

Physiological models of cardiovascular regulation and RSA have focused on the neural, autonomic, and baroreflex mechanisms responsible for cardiorespiratory interactions. Early control-theoretic descriptions of cardiovascular regulation [1,2] were followed by more detailed physiological models incorporating autonomic control, respiratory influences on heart-rate variability, and baroreflex regulation [72,73,74,75]. These studies clarified the physiological pathways contributing to RSA and provided mechanistic interpretations of observed cardiorespiratory dynamics.

Broader physiological analyses have emphasised the closed-loop nature of cardiorespiratory regulation and the multiple respiratory, autonomic, and baroreflex pathways contributing to cardiorespiratory coupling [76,77,78].

A complementary modelling approach represents respiration and cardiac activity as interacting oscillators. The cardiovascular system has been interpreted as a hierarchy of coupled oscillatory dynamics spanning multiple temporal scales [18]. Synchronisation between heartbeat and respiration was demonstrated experimentally by Schäfer et al. [79], motivating the development of oscillator-based descriptions of cardiorespiratory coordination [17,19].

Among the early oscillator descriptions, Lotrič and Stefanovska [7] analysed synchronisation and modulation in the human cardiorespiratory system using coupled phase oscillators, demonstrating that cardiorespiratory coordination can be interpreted in terms of dynamical synchronisation phenomena rather than solely through statistical measures of variability. Recent work has further suggested that synchronisation in the cardiorespiratory system may be related to dissipation and energetic exchange processes, offering a complementary thermodynamic perspective on oscillatory coordination [39]. Subsequent models reproduced synchronisation, entrainment, and RSA dynamics using coupled oscillators or integrate-and-fire formulations [80,81,82,83]. These studies established synchronisation as a useful dynamical concept for understanding cardiorespiratory organisation.

A further development has been the reconstruction of interaction structure directly from physiological recordings. Coupling functions, dynamical Bayesian inference, state-space methods, and information-theoretic approaches have provided quantitative descriptions of directionality, phase dependence, and temporal variability in cardiorespiratory interactions [16,55,78,84,85]. In particular, population studies demonstrated systematic age-related changes in the organisation of cardiorespiratory interactions across healthy individuals [16]. These approaches showed that cardiorespiratory coupling is inherently time dependent and varies across physiological states and ageing.

Table 1 provides a comparative overview of the main physiological, data-driven, and modelling approaches used to investigate cardiorespiratory interactions, highlighting how the present model relates to each of them.

The proposed formulation complements existing physiological, signal-processing, and data-driven approaches by focusing on the dynamical stability of synchronisation under explicit time-dependent modulation. Unlike previous oscillator formulations, it is non-autonomous from the outset, with time dependence incorporated directly into the governing equations through frequency modulation. The model therefore represents cardiorespiratory interaction as an evolving dynamical process rather than a stationary coupling structure. Synchronisation is characterised through time-dependent attractors and finite-time stability, while physiological observations and coupling functions inferred from data provide reference points for comparison with measured cardiorespiratory dynamics.

## 6. Discussion

The present work develops a non-autonomous oscillator formulation for describing RSA as a dynamically modulated interaction between cardiac and respiratory rhythms, aiming to reinterpret cardiorespiratory coupling as a continuously evolving dynamical process rather than a static interaction encoded by fixed coupling functions.

Earlier coupled oscillator models treated the cardiorespiratory interaction as the dynamics of self-sustained oscillators under autonomous or weakly forced conditions [17,19]. These descriptions successfully reproduced the phase-locking and Arnold tongue structures, but generally assumed fixed coupling properties. More recent data-driven studies have shown that cardiorespiratory coupling is time-dependent, motivating time-resolved inference of interaction functions [44,60], without an explicit dynamical description of coupling evolution.

In parallel, non-autonomous and chronotaxic oscillator theory provides a basis in which stability is defined through tracking time-dependent attractors [31,32,33,35,36,38]. The present model extends these ideas beyond the slow-modulation regime commonly considered in earlier analyses, where the modulation frequency is assumed to be much smaller than the intrinsic oscillator frequency. In the simulations presented here, the modulation frequency $\omega_{m}=0.5\pi$ is not asymptotically separated from the driving frequency $\omega_{1}=6$, so the adiabatic approximation is no longer applicable and the dynamics must be analysed numerically.

Here, we extend this perspective by treating the interaction structure itself as a dynamical quantity. Physiological regulation is therefore reflected not only in state evolution but also in the temporal organisation of coupling. RSA is therefore interpreted as a structured form of respiration-driven phase modulation rather than as a perturbation of an autonomous cardiac oscillator.

A key implication is that physiological variability is not external to the system but arises from explicit time dependence in both the intrinsic dynamics and interaction structure. This is supported by the comparison between participants, where the younger subject exhibits stronger temporal variability in coupling strength, while the older subject shows a more stationary interaction profile. Importantly, this difference reflects not a change in mean coupling strength, but a reduction in the temporal degrees of freedom of the interaction.

This suggests that ageing is associated with reduced variability of coupling rather than with weaker coupling per se, implying a loss of dynamical flexibility in cardiorespiratory coordination. This interpretation is consistent with earlier studies showing that ageing is accompanied by a general reduction in cardiovascular variability and complexity [63], as well as with previous analyses of age-dependent changes in the cardiorespiratory interaction and synchronisation structure [16].

From a modelling perspective, this motivates the introduction of explicitly time-dependent coupling functions

$$f_{c}\left(\phi_{h},\phi_{r},t\right),\;g_{c}\left(\phi_{h},\phi_{r},t\right),$$

allowing the interaction structure to evolve on its own intrinsic time scale. More generally, the present results suggest that physiological function is best understood in terms of time-dependent interactions rather than isolated subsystem dynamics. The most informative signatures of regulation are therefore contained, not in average oscillator properties, but in the temporal structure of their coupling, which encodes adaptive control across multiple time scales.

## 7. Summary and Conclusions

The cardiorespiratory interaction is better represented as a time-varying dynamical process rather than as a system with static coupling. The coupling strength varies in time, with reduced temporal variability observed in older subjects. These findings indicate that physiological regulation is reflected in an evolving interaction structure rather than fixed coupling parameters, and that the temporal variability of coupling constitutes a measurable characteristic of the dynamics.

Several extensions follow naturally. The assumption of sinusoidal coupling may be relaxed to allow more general forms of nonlinear interaction. Self-coupling effects, neglected here, may contribute to intrinsic regulation of cardiac and respiratory rhythms. In addition, coupling strength should be treated as a time-varying quantity inferred directly from data rather than as a fixed parameter.

Finally, future developments may include extension to additional physiological oscillations, such as vascular myogenic, neurogenic, and endothelial rhythms, as well as to the construction of personalised models inferred from individual recordings, enabling direct quantification of time-dependent intrinsic dynamics and interaction structure across subjects and conditions.

## Figures and Tables

**Table 1:** Data-driven and model-driven approaches used to assess cardiorespiratory interactions.

Approach	Primary Quantity	Main Contribution	Relation to the Present Model
RSA and spectral indices [64,65,67,71]	RSA magnitude, spectral power	Quantify respiratory modulation of heart-rate variability	Do not provide a dynamical description of evolving interactions or stability
Transfer-function and coherence analysis [66]	Gain, phase, coherence	Characterise frequency-dependent relationships between respiration and heart-period variability	Primarily linear and stationary descriptions
Physiological control models [1,2,72,73]	Mechanistic cardiovascular variables	Represent neural, mechanical, and autonomic regulation	Focus on physiological pathways rather than dynamical stability of synchronisation
Physiological perspectives on cardiorespiratory coupling [76,77]	Physiological interaction mechanisms	Synthesise physiological mechanisms underlying cardiorespiratory coupling	Provide physiological context for interpreting model dynamics
Information-theoretic and state-space methods [56,84]	Directionality, predictability, information transfer	Quantify interaction strength, directionality, and causal asymmetry	Do not explicitly model the underlying dynamical system
Oscillator and synchronisation models [7,19,39,47,79,80,81]	Phase locking, entrainment, phase coherence	Provide dynamical descriptions of synchronisation and rhythmic coordination	Typically formulated as autonomous or weakly perturbed systems
Coupling-function and inference approaches [16,55,84,85]	Phase-dependent interaction functions	Reveal interaction structure, directionality, and time variability directly from data	Interaction functions are inferred but not embedded in a non-autonomous stability description
Present work	Time-dependent attractors, finite-time Lyapunov exponents, evolving synchronisation	Models RSA as a non-autonomous system with explicitly time-dependent modulation and stability	Provides a dynamical description complementary to existing measures rather than replacing them

## Data Availability

Datasets for the participants included in the study are available on PURE (https://doi.org/10.17635/lancaster/researchdata/747 (accessed on 7 June 2026)). Specifically, data from subjects no. 229 (22 year-old male) and no. 11 (72 year-old male) were analysed in this study.

## Abbreviations

The following abbreviations are used in this manuscript:

RSA	Respiratory Sinus Arrhythmia
HRV	Heart Rate Variability
ECG	Electrocardiogram
FTLE	Finite-Time Lyapunov Exponent(s)
CWT	Continuous Wavelet Transform
DBI	Dynamical Bayesian Inference
